# From Composition to Function: Lime Essential Oil (*Citrus aurantifolia*) and (*R*)-(+)-Limonene and Their Impact on Rumen Microbiota, Fermentation, Methane Emission and Blood Metabolic Parameters in Dairy Cows

**DOI:** 10.3390/molecules31142403

**Published:** 2026-07-08

**Authors:** Natalia Pachura-Hanusek, Sylwia Banaszkiewicz, Kamila Lewandowska, Anna Burek, Antoni Szumny, Aleksandra Tabiś, Jacek Bania, Robert Kupczyński

**Affiliations:** 1 Department of Environment Hygiene and Animal Welfare, The Faculty of Biology and Animal Science, Wrocław University of Environmental and Life Sciences, Chełmońskiego St. 38c, 50-375 Wrocław, Poland; natalia.pachura@upwr.edu.pl (N.P.-H.); kamila.lewandowska@upwr.edu.pl (K.L.); anna.burek@upwr.edu.pl (A.B.); 2 Department of Food Hygiene and Consumer Health Protection, Wrocław University of Environmental and Life Sciences, Norwida St. 31, 50-375 Wrocław, Polandaleksandra.tabis@upwr.edu.pl (A.T.); jacek.bania@upwr.edu.pl (J.B.); 3 Department of Biocatalysis and Food Chemistry, Faculty of Biotechnology and Food Science, Wrocław University of Environmental and Life Sciences, Norwida St. 25, 50-375 Wrocław, Poland; antoni.szumny@upwr.edu.pl

**Keywords:** lime essential oil, (*R*)-(+)-Limonene, oils GC–MS analysis, rumen fermentation, volatile fatty acids, methanogenesis, rumen microbiome, 16S rRNA gene sequencing, dairy cow

## Abstract

In this study, we characterized the chemical composition of lime essential oil (*Citrus aurantifolia*) by GC–MS and evaluated the effects of lime essential oil and its major constituent, (*R*)-(+)-limonene, on rumen fermentation, methane production, microbial community structure and metabolic responses in dairy cows. In vitro assays were used to establish effective doses for in vivo application. In vivo supplementation with lime essential oil on day 14 significantly increased ruminal pH (6.96 vs. 6.66; *p* < 0.05), total VFA concentration (4.84 vs. 4.11 mg mL^−1^; *p* < 0.05), serum glucose (3.92 vs. 3.63 mmol L^−1^; *p* < 0.05) and bicarbonate concentration (26.75 vs. 22.35 mmol L^−1^; *p* < 0.05), indicating improved fermentation efficiency and buffering capacity. Metabolic profiling revealed elevated glucose and reduced non-esterified fatty acids, suggesting enhanced energy utilization and decreased lipid mobilization, without adverse effects on liver enzymes (AST, ALT, GGT), lipid profile or acid–base balance. Rumen microbiota remained stable and was dominated by Bacteroidota and Bacillota; no significant changes occurred in alpha or beta diversity. (*R*)-(+)-limonene selectively reduced the abundance of Methanobacteriota (*p* = 0.04). Methane concentrations were not significantly affected. Both additives exerted beneficial, dose-dependent effects on rumen function and host metabolism and show promise as natural feed additives in dairy cows.

## 1. Introduction

The “One Health” concept, which recognizes the interconnections between human, animal, and environmental health, is becoming a key subject of focus in sustainable animal production [1]. In the face of global challenges related to greenhouse gas emissions, increasing attention is being paid to sustainable development strategies in animal production, including the reduction in enteric methane emissions from dairy cows [2,3]. In response to growing concerns regarding greenhouse gas emissions and the sustainability of livestock production, considerable efforts have been directed toward developing nutritional strategies that improve rumen efficiency while reducing the environmental impact of ruminant systems [4,5]. One promising approach is the use of natural feed additives as alternatives to synthetic compounds, including antibiotics, for improving animal health and the quality of animal-derived products [4,5]. Among these additives, essential oils (EOs) derived from aromatic plants have attracted significant scientific interest due to their antimicrobial properties and their potential to modulate the rumen microbiome’s fermentation processes [4,5,6].

Studies have shown that EOs can selectively inhibit the growth of certain microorganisms in rumen fluid, reduce methane production, and influence the profile of volatile fatty acids (VFAs), which may simultaneously contribute to improved nutrient utilization efficiency [7,8,9]. In vitro experiments have shown that EO mixtures exhibit synergistic effects, altering the structure of the microbiota and rumen metabolism [10,11,12]. Furthermore, in vivo studies have suggested that supplementation with EOs or phytobiotics may improve milk yield, reduce methane emissions, and modulate the rumen microbiome, thereby positively affecting N utilization and overall feed efficiency [3,13,14]. The degradation of various feed components occurs through the interaction of the microbiome, resulting primarily in the production of acetate, propionate, butyrate, hydrogen (H_2_), carbon dioxide (CO_2_), and ammonia (NH_3_). While the VFAs produced in this way are the primary energy source for ruminants, certain byproducts, such as CO_2_ and H_2_, are also produced, which are then converted into CH_4_ by certain methanogens and archaea [15,16]. CH_4_ production deprives the ruminant of carbon resources and results in energy loss (13.3 Mcal kg^−1^ CH_4_), leading to low feed conversion efficiency [15,17]. Methane emissions in ruminants are highly variable and are largely determined by the structure and functional characteristics of the rumen microbial ecosystem. Variations in microbial community composition and metabolic pathways influence hydrogen flow within the rumen, which in turn affects methanogenic activity and CH_4_ production [18]. Further reductions in methane emissions can be achieved through dietary means without negatively affecting the animal [13,19,20,21]. A review by Palangi and Lackner indicates that natural compounds added to cows’ diet can reduce methane emissions by 10–30% [20].

Among plant essential oils, lime essential oil (*Citrus aurantifolia*) and other citrus essential oils have attracted particular interest [2,22,23,24], as these compounds exhibit antimicrobial properties and can selectively inhibit the growth of pathogenic microorganisms in the rumen while also influencing fermentation and nitrogen metabolism [22,25,26]. Citrus essential oils, including that from lime (*Citrus aurantifolia*), are a complex mixture of volatile compounds dominated by monoterpenes, sesquiterpenes, alcohols, aldehydes, esters, and flavonoids [2,24]. Depending on the citrus species and extraction conditions, the limonene content in the oil can range from 30% to over 70% of the total oil volume [27,28].

Although in vitro studies have found that citrus essential oils, including limonene, can reduce methane production and modulate volatile fatty acid profiles, there is still a lack of studies directly evaluating the effect of lime essential oil on cows’ diets and its effects on rumen bacterial metagenomics [23,24,27]. Given the complex composition of essential oils, evaluating both lime essential oil and its major constituent, (*R*)-(+)-limonene, provides an opportunity to distinguish the effects of the whole phytogenic mixture from those of a single bioactive compound. Such an approach may improve our understanding of the mechanisms through which citrus-derived compounds influence rumen fermentation, methanogenesis, and microbial community structure.

In this study, our aims were to characterize the chemical composition of lime essential oil and (*R*)-(+)-limonene, and to assess their antimicrobial activity against selected rumen microorganisms through preliminary in vitro experiments, including the determination of minimum inhibitory concentrations (MICs) and their effects on fermentation parameters and methane production. Subsequently, we aimed to evaluate the impact of these compounds in vivo in dairy cows, with a particular focus on rumen fermentation, methane emissions, and the metagenomic profiling of rumen bacterial communities. We hypothesize that, due to their chemical composition, lime essential oil and (*R*)-(+)-limonene exert differential antimicrobial effects on rumen microbiota, leading to shifts in microbial community structure and fermentation patterns.

## 2. Results

### 2.1. Effects of Essential Oils on Cultures of Rumen Microorganisms

To determine the minimum inhibitory concentrations (MICs) for four key bacteria of the natural rumen microflora, MIC values (Table 1) were established for limonene-rich plants and limonene enantiomers. We aimed to identify growth-promoting (GS) concentrations while maintaining MIC values as low as economically and technologically feasible, avoiding excessive supplementation levels for practical ruminant nutrition applications. Among the analyzed citrus oils characterized by high limonene content, orange oil (*Citrus sinensis*) exhibited high MIC values (1600 ppm) against *Butyrivibrio fibrisolvens*, *Prevotella albensis*, and *Lactobacillus delbrueckii*, and 3200 ppm against *Streptococcus bovis*. However, it effectively stimulated growth at 200 ppm for *Butyrivibrio fibrisolvens* and at 25 ppm for *Prevotella albensis*. High MIC values were also observed for three strains—*Butyrivibrio fibrisolvens*, *Prevotella albensis* (1600 ppm), and *Streptococcus bovis* (>6400 ppm)—while a low MIC value of 400 ppm for *Lactobacillus delbrueckii* was obtained after the application of lemon oil. *Citrus aurantifolia* demonstrated optimal characteristics, achieving the lowest GS values (25–50 ppm) alongside MIC across *Butyrivibrio fibrisolvens* (800 ppm), *Prevotella albensis* (200 ppm), and *Lactobacillus delbrueckii* (200 ppm), with *Streptococcus bovis* (1600 ppm) indicating effective growth stimulation within safe dosage limits. The lowest MIC values were obtained after the application of *Cymbopogon citratus*, namely *Butyrivibrio fibrisolvens* (200 ppm), *Prevotella albensis* (200 ppm), *Butyrivibrio fibrisolvens* (1600 ppm), *Prevotella albensis* (1600 ppm), and *Streptococcus bovis* (1600 ppm). The other essential oils derived from rosemary and peppermint, despite their high MIC values, did not exhibit growth stimulation. Among the pure enantiomeric compounds, both exhibited high MIC values against *Butyrivibrio fibrisolvens* (3200 ppm for (*S*)-(−)-limonene; 4000 ppm for (*R*)-(+)-limonene) and *Streptococcus bovis* (6400 ppm for both (*S*)-(−)- and (*R*)-(+)-limonene), as well as *Lactobacillus delbrueckii* (6400 ppm for (*S*)-(−)-limonene; 1600 ppm for (*R*)-(+)-limonene) and *Prevotella albensis* (1600 ppm for (*R*)-(+)-limonene). In contrast, a low MIC value was observed for *Prevotella albensis* after the addition of (*S*)-(−)-limonene (400 ppm).

### 2.2. GC-MS Characterization of Citrus aurantifolia Essential Oil

GC-MS analysis of lime essential oil revealed a complex composition comprising 70 volatile compounds (Table 2). The obtained profile clearly indicates a mixture dominated by terpenoid constituents, with a strong predominance of monoterpene hydrocarbons. Among all identified compounds, limonene was found to be the major component, accounting for 46.57% of the total composition, confirming its dominant role in determining the chemical characteristics of the oil.

In addition to limonene, several other monoterpene hydrocarbons were present at relatively high levels, including β-pinene (12.01%), γ-terpinene (10.71%), and *p*-cymene (6.50%), collectively contributing to the typical terpene-rich profile of citrus essential oils. Other monoterpenes, such as α-pinene, sabinene, and myrcene, were detected, although in lower concentrations, generally below 3%. Oxygenated monoterpenes were also detected, although they were present at relatively low levels. These included linalool (0.22%), terpinen-4-ol (0.13%), and citral isomers, such as neral (1.08%) and geranial (1.73%). Despite their relatively low concentrations, these compounds may contribute significantly to the biological activity of the oil due to their higher chemical reactivity. Among them, β-bisabolene (2.75%) and *trans*-α-bergamotene (1.86%) were the most abundant representatives, while the remaining compounds from this group occurred in trace amounts.

### 2.3. Effects of Lime Essential Oil and (R)-(+)-Limonene on In Vitro Fermentation

Supplementation with lime essential oil and (*R*)-(+)-limonene modulated in vitro ruminal fermentation in a dose- and time-dependent manner (Table 3). After 4 h of incubation, none of the treatments significantly affected the medium’s pH (*p* = 0.06). However, after 24 h, a significant treatment effect emerged (*p* = 0.03), with the highest pH value observed in the 50 ppm (*R*)-(+)-limonene group (6.80 vs. 6.55 in control).

After 24 h of incubation, NH_3_ concentration ranged from 217.5 mg L^−1^ in the 50 ppm lime EO treatment to 282.0 mg L^−1^ in the 25 ppm (*R*)-(+)-limonene treatment (*p* = 0.018), representing a 23% reduction in the 50 ppm lime EO group compared with the highest limonene treatment. This suggests a selective inhibition of deaminative bacteria by the complete essential oil matrix. Similarly, propionate concentration increased significantly from 0.365 mg mL^−1^ in the control to 0.430 mg mL^−1^ with 50 ppm lime EO (+18%; *p* < 0.05) and to 0.429 mg mL^−1^ with 50 ppm (*R*)-(+)-limonene. The acetate–propionate ratio was lowest in the 25 ppm lime EO group (1.47), indicating a shift toward more energetically efficient fermentation pathways that favor propionate production.

Total VFA production showed numerical but non-significant variation: the 25 ppm lime EO group yielded the highest TVFA after both 4 h and 24 h, whereas 25 ppm (*R*)-(+)-limonene produced the lowest values, suggesting that the complete oil at a moderate dose supports overall fermentative activity better than the isolated monoterpene. Methane production after 24 h was numerically 17–22% lower in all supplemented groups compared with the control (lowest value in 25 ppm (*R*)-(+)-limonene), although differences did not reach statistical significance. These results confirm the dose-dependent modulation of fermentation end-products and support the selection of 50 ppm as the most effective and biologically relevant level for subsequent in vivo application.

### 2.4. Effects of Lime Essential Oil and (R)-(+)-Limonene Supplementation on Rumen Fermentation—In Vivo Study

Supplementation did not alter ruminal pH during the first week, confirming the absence of acute effects on the acid–base balance (Table 4). However, on day 14, a clear time-dependent response was observed: lime EO increased ruminal pH to 6.96 compared with 6.66 in the control (+0.30 units; *p* < 0.05) and 6.54 in the (*R*)-(+)-limonene group. This rise in pH after two weeks of supplementation suggests an improved ruminal buffering capacity and reduced accumulation of fermentation acids following microbial adaptation to the phytogenic additive. The lack of a similar long-term effect in the limonene-only group indicates that synergistic interactions among multiple terpenoids present in the complete essential oil are required for stable modulation of the ruminal environment.

Ammonia nitrogen dynamics differed markedly on day 3: lime EO reduced NH_3_-N concentration, whereas (*R*)-(+)-limonene increased it, pointing to divergent short-term effects on microbial nitrogen metabolism and amino acid deamination. These differences disappeared by days 7 and 14. Total VFA concentration followed a biphasic pattern: on day 3, (*R*)-(+)-limonene significantly elevated TVFA, while lime EO reduced it. By day 14, the response reversed: lime EO produced the highest TVFA concentration (4.84 mg mL^−1^), representing an 18% increase over the control (4.11 mg mL^−1^; *p* < 0.05). Acetate concentration mirrored this pattern, reaching its peak in the lime EO group on day 14 (*p* < 0.05), consistent with enhanced fiber fermentation after microbial adaptation. These temporal shifts demonstrate that lime EO requires approximately two weeks to exert its full stimulatory effect on fermentative activity, whereas isolated limonene elicits a more rapid but transient response.

Methane concentrations in rumen gas gradually became comparable across treatments. Importantly, (*R*)-(+)-limonene selectively reduced the relative abundance of Methanobacteriota (*p* = 0.04) without affecting overall alpha or beta diversity, indicating targeted rather than broad-spectrum suppression of methanogenic populations.

### 2.5. Effects of Lime Essential Oil and (R)-(+)-Limonene Supplementation on Blood Parameters

The effects of the applied treatment are summarized in Table 5. The most pronounced changes were observed in carbohydrate metabolism: on day 14 of the experiment, a statistically significant increase in glucose concentration was recorded in the group receiving limonene (3.92 ± 0.09 mmol L^−1^) compared to the control group (3.63 ± 0.15 mmol L^−1^) and the limonene R group (3.38 ± 0.06 mmol L^−1^) (*p* < 0.05). At the same time, NEFA levels were reduced in the lime EO group. The analysis of key hepatic enzymes (AST, ALT, GGT) did not reveal any statistically significant differences (*p* > 0.05) between the control group and the groups receiving limonene and (*R*)-(+)-limonene at any timepoint. Supplementation did not significantly affect the lipid profile of the animals studied. Total cholesterol, HDL and LDL fractions, and triglyceride concentrations did not differ significantly between groups at any sampling point. Total protein showed a significant group effect (*p* < 0.04): over the course of the experiment, TP and albumin in the lime EO group exhibited an increasing tendency, whereas an opposite trend was observed in the (*R*)-(+)-limonene group. No significant effects of treatment or time were found for total antioxidant status (TAS) or LA.

The effects of supplementation on serum biochemical parameters are presented in Table 5. The most pronounced changes were observed for glucose concentration. Significant effects of time (*p* = 0.02) and the TG × T interaction (*p* < 0.01) were detected. On day 14, cows supplemented with lime EO showed significantly higher glucose concentration compared with both the control and (*R*)-(+)-limonene groups (3.92 vs. 3.63 and 3.38 mmol L^−1^, respectively; *p* < 0.05).

While no significant treatment group or interaction effects were observed for NEFA concentration, a significant effect of time was detected. Numerically lower NEFA values were observed in the lime EO group on day 14 compared with the remaining groups.

Supplementation did not significantly affect serum lipid profile parameters, including total cholesterol, HDL, LDL, and triglycerides, as no significant effects of treatment, time, or interaction were observed. Similarly, creatinine concentration remained stable throughout the experiment. No statistically significant differences were detected for hepatic enzyme activities (AST, ALT, and GGT), indicating that supplementation did not affect liver-related biochemical parameters during the experimental period.

Total protein showed a significant treatment group effect (*p* < 0.05), whereas no significant effects of time or TG × T interaction were observed. Albumin concentration was not significantly affected by supplementation or sampling time. Numerically higher TP and albumin values were observed in the lime EO group during the experiment, whereas lower values were generally noted in the (*R*)-(+)-limonene group. No significant effects of treatment, time, or interaction were observed for total antioxidant status (TAS) or lactate concentration (*p* > 0.05), although lactate concentration differed between groups at day 0. Likewise, no significant effects of treatment group, time, or TG × T interaction were observed for lactate concentration throughout the experimental period.

**Figure 1:**
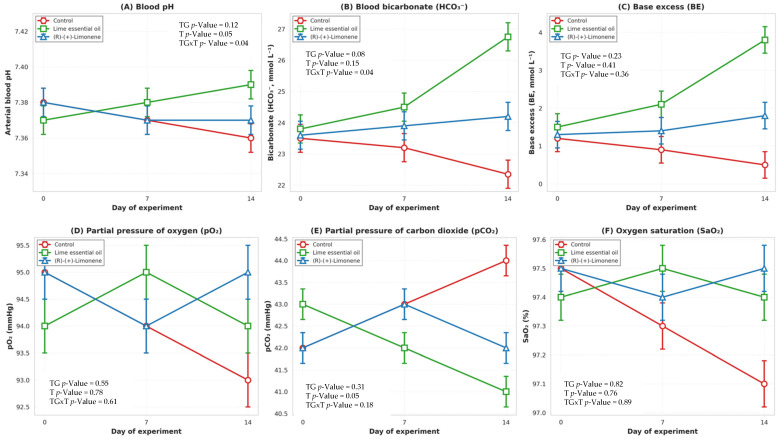
Acid-base status in arterial blood of experimental groups supplemented with lime essential oil and (*R*)-(+)-limonene. (**A**) Arterial blood pH; (**B**) Blood bicarbonate (HCO_3_^−^); (**C**) Base excess (BE); (**D**) Partial pressure of oxygen (pO_2_); (**E**) Partial pressure of carbon dioxide (pCO_2_); (**F**) Oxygen saturation (SaO_2_). Data are presented as means ± SEM. *p*-values are presented for the main effects of treatment group (TG), time (T), and the treatment group × time interaction (TG × T).

Blood acid–base parameters are presented in Figure 1. Arterial blood pH showed only minor variations between groups during the experimental period. Significant effects of time (*p* < 0.05) and TG × T interaction (*p* = 0.04) were observed for blood pH, whereas no significant treatment group effect was detected. No significant effects of treatment group, time, or TG × T interaction were observed for pO_2_ or SaO_2_ (*p* < 0.05). For pCO_2_, a significant effect of time was detected (*p* = 0.05), whereas treatment group and interaction effects remained non-significant. Bicarbonate concentration (HCO_3_^−^) showed a significant TG × T interaction effect (*p* < 0.04), whereas the treatment group effect showed only a tendency toward significance (*p* < 0.08). On day 14, cows supplemented with lime EO exhibited significantly higher HCO_3_^−^ concentration compared with both the control and (*R*)-(+)-limonene groups (26.75 vs. 22.35 and 24.60 mmol L^−1^, respectively; *p* < 0.05). Base excess (BE) was not significantly affected by treatment group, time, or interaction in the overall model (*p* < 0.05). Nevertheless, on day 14, higher BE values were observed in the lime EO group compared with the control and (*R*)-(+)-limonene groups.

### 2.6. Microbiome Analysis and Taxonomic Composition

High-throughput sequencing of the V3–V4 region of the 16S rRNA gene yielded an average of 289,381 (±54,167 SD) raw read pairs per sample. After quality filtering, 91.66% of reads were retained. Subsequent denoising, merging, and chimera removal using the DADA2 pipeline resulted in 196,240 (±44,024 SD) high-quality sequences per sample, corresponding to 67.67% of the initial input.

**Figure 2:**
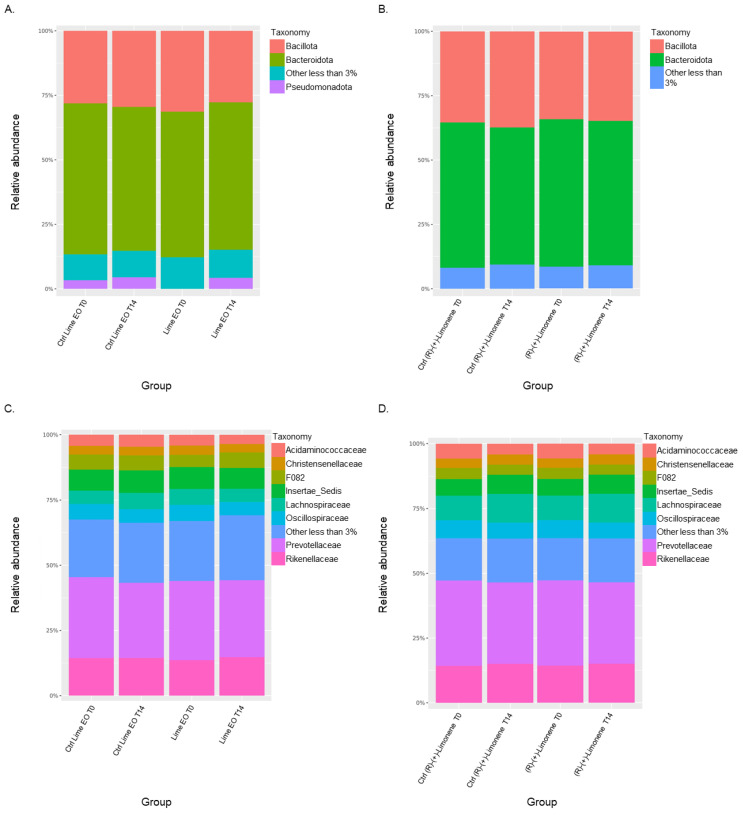
Relative abundance of dominant microbial taxa across experimental groups and timepoints. (**A**) Relative abundance of bacterial and archaeal phyla in the lime EO experiment. (**B**) Relative abundance of bacterial and archaeal phyla in the (*R*)-(+)-limonene experiment. (**C**) Relative abundance of dominant bacterial families in the lime EO experiment. (**D**) Relative abundance of dominant bacterial families in the (*R*)-(+)-limonene experiment. Stacked bar plots represent mean relative abundance (%) in control and treatment groups at baseline (T0) and after 14 days of incubation (T3). Taxa with relative abundance below 3% were grouped as “Other less than 3%.”.

Taxonomic classification rates were high across all ranks, reaching 99.80% at the phylum level and 99.51% at the family level. Although genus- and species-level assignments were 98.69% and 56.07%, respectively, these were excluded from downstream analyses due to the limited resolution of short-read 16S rRNA sequencing. The microbial community was dominated by Bacteroidota (56.2 ± 3.9%) and Bacillota (32.8 ± 5.2%), together accounting for over 88% of all classified reads. Other phyla detected at lower abundance included Pseudomonadota (3.0 ± 2.3%), Patescibacteria (2.0 ± 0.6%), and Verrucomicrobiota (2.0 ± 0.6%) (Figure 2A,B).

At the family level, Prevotellaceae (30.3 ± 4.0%) and Rikenellaceae (14.3 ± 2.3%) were the most abundant taxa, followed by Lachnospiraceae (9.2 ± 2.2%) and Oscillospiraceae (6.5 ± 1.4%). A proportion of reads was assigned to unclassified lineages, including Incertae Sedis (8.0 ± 1.2%) and F082 (6.0 ± 0.7%) (Figure 2C,D).

No significant differences (*p* > 0.05) were observed in the relative abundance of dominant bacterial phyla between T0 and T14 in either experimental trial, indicating the overall stability of the core microbiome. In the (*R*)-(+)-limonene experiment, a significant reduction in the archaeal phylum Methanobacteriota was observed, decreasing from 0.41 ± 0.06% at T0 to 0.16 ± 0.06% at T14 (*p* = 0.04), corresponding to a reduction of more than 60%. Detailed results for bacterial and archaeal taxa showing significant differences in relative abundance between experimental groups and timepoints are provided in Supplementary Table S1.

#### Alpha and Beta Diversity

Alpha Diversity: To evaluate within-sample microbial diversity, five alpha diversity metrics were calculated: Observed ASVs and Chao1 (richness estimators), and the Shannon, Simpson, and Inverse Simpson indices (diversity and evenness measures). Summary alpha diversity metrics for all experimental groups are presented in Table 6.

In the lime EO experiment, alpha diversity indices were stable across groups and timepoints. No significant differences were observed between T0 and T3 for any diversity metric (*p* > 0.05, Welch’s *t*-test). A significant difference was detected between Ctrl lime EO T0 and lime EO T14 for Observed ASVs and Chao1 (*p* = 0.012 and *p* = 0.011, respectively), as summarized in Table 6.

In the (*R*)-(+)-limonene experiment, no significant changes in alpha diversity were observed within the treated group over time ((*R*)-(+)-limonene T0 vs. (*R*)-(+)-limonene T14; *p* > 0.05). In contrast, the control group showed a modest but significant temporal shift in richness over time (T0 vs. T3), reflected by the Observed ASVs and Chao1 indices (*p* = 0.045 and *p* = 0.044, respectively; Table 6).

No significant differences were observed for Shannon, Simpson, or Inverse Simpson indices across any comparisons in either experiment (*p* > 0.05), indicating stable diversity and evenness across all groups (Table 6).

Detailed per-sample alpha diversity values are provided in Supplementary Table S2.

Beta Diversity: To evaluate differences in microbial community structure, beta diversity was assessed using Bray–Curtis dissimilarity matrices based on ASV profiles. The statistical significance of differences in the microbial community structure was assessed using PERMANOVA.

**Figure 3:**
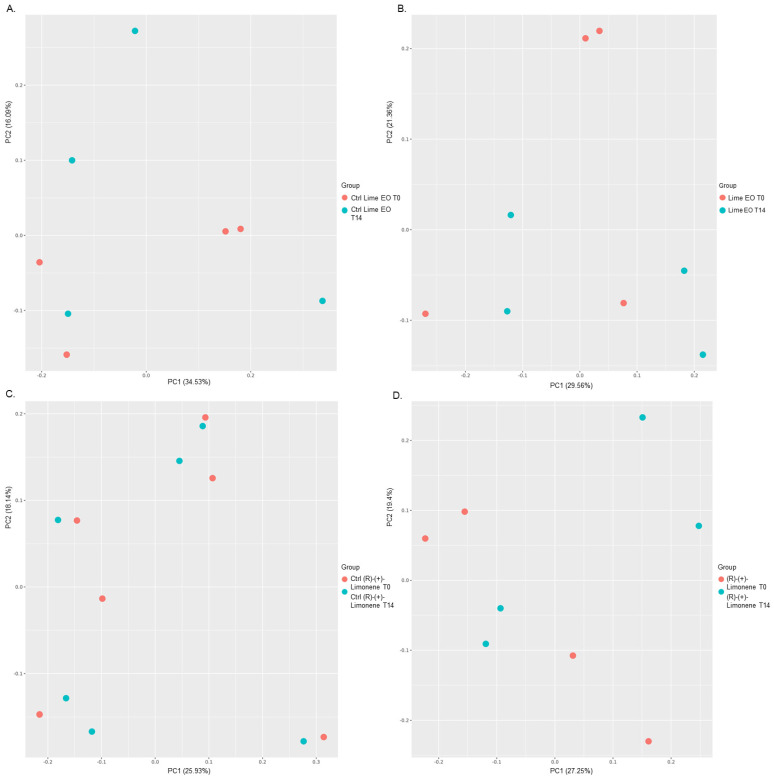
Principal Coordinate Analysis (PCoA) of microbial community structure across experimental groups and timepoints. (**A**) Lime EO experiment, control group (Ctrl lime EO T0 and Ctrl lime EO T14). (**B**) Lime EO experiment, treatment group (lime EO T0 and lime EO T14). (**C**) (*R*)-(+)-limonene experiment, control group (Ctrl (*R*)-(+)-limonene T0 and Ctrl (*R*)-(+)-limonene T14). (**D**) (*R*)-(+)-limonene experiment, treatment group ((*R*)-(+)-limonene T0 and (*R*)-(+)-limonene T14). PCoA plots were generated based on Bray–Curtis dissimilarity and visualize differences in microbial community composition between samples. Each point represents an individual sample.

In the lime EO experiment, no significant differences in community composition were observed between timepoints within the treatment group (lime EO T0 vs. lime EO T14: Pseudo-F = 0.79, R^2^ = 0.116, *p* = 0.747) or within the control group (Ctrl lime EO T0 vs. Ctrl lime EO T14: Pseudo-F 0.96031, R2 = 0.138, *p* = 0.545). Similarly, no significant differences were detected between treatment and control groups at the end of the supplementation period (Ctrl lime EO T14 vs. lime EO T14: Pseudo-F = 0.69, R^2^ = 0.103, *p* = 0.857). Consistent with these results, PCoA ordination did not reveal distinct clustering of samples according to treatment or timepoint, with substantial overlap observed across all groups (Figure 3A,B).

A similar pattern was observed in the (*R*)-(+)-limonene experiment. No significant differences in beta diversity were detected between timepoints within the treatment group ((*R*)-(+)-limonene T0 vs. (*R*)-(+)-limonene T14: Pseudo-F = 0.71, R^2^ = 0.105, *p* = 0.898) or within the control group (Ctrl (*R*)-(+)-limonene T0 vs. Ctrl (*R*)-(+)-limonene T14: *p* = 0.375). Comparisons between treatment and control groups at the final timepoint also showed no significant differences (Ctrl (*R*)-(+)-limonene T14 vs. (*R*)-(+)-limonene T14: Pseudo-F = 0.82, R^2^ = 0.093, *p* = 0.739). PCoA visualization confirmed the absence of clear group separation, indicating the high similarity of the microbial community structure across all experimental conditions (Figure 3C,D).

## 3. Discussion

Lime essential oil (*Citrus aurantifolia*) and its main constituent, limonene, exhibit significant biological activity, which is consistent with previous literature reports on citrus essential oils [29,30,31,32,33]. Due to the growing interest in natural products and their wide range of applications, a better understanding of their biological mechanisms of action is essential to identify new uses, particularly in animal health prevention and as functional feed additives. Some of these natural products represent an effective alternative to synthetic additives commonly used in livestock production [34,35,36]. A key factor determining the biological efficacy of essential oils is their chemical composition, which influences both the direction and magnitude of their effects on animal metabolism and microbiota, thereby shaping their potential as functional feed additives.

The GC–MS analysis performed in the present study confirmed that lime essential oil is dominated by monoterpene hydrocarbons, with limonene as the principal constituent. This finding is consistent with previous reports on citrus essential oils, in which limonene typically represents the major component, with its content varying widely depending on species, origin, and extraction conditions. In the literature, limonene has been reported to range from approximately 25% to over 90% of the total composition in citrus-derived essential oils [37,38,39].

Importantly, essential oils and their bioactive components, including limonene, have also been evaluated in vivo in ruminants. In studies conducted on sheep, supplementation with blends of essential oils containing citrus components rich in limonene did not significantly affect methane production, rumen fermentation parameters, or short-chain fatty acid concentrations, although slight changes in nutrient digestibility were observed. These findings suggest that the effectiveness of essential oils in modulating rumen function may depend on the composition of the blend and the applied dose [40].

In contrast, more pronounced effects have been reported in cattle. For example, supplementation with orange essential oil in heifers resulted in a significant reduction in enteric methane emissions without major alterations in rumen fermentation parameters or feed intake, indicating that citrus essential oils rich in limonene may selectively inhibit methanogenesis while maintaining overall fermentation stability [41].

Similarly, studies conducted on steers demonstrated that orange essential oil, containing high levels of limonene (approximately 88%), can influence rumen fermentation by increasing short-chain fatty acid production and reducing methane output, thereby improving the energetic efficiency of fermentation. Notably, these effects were dose-dependent, with moderate inclusion levels showing the greatest reduction in methane production [42]. The tested additives did not significantly affect the pH of the rumen fluid, confirming their in vivo safety and the lack of risk of acidosis [43]. The lower ammonia concentrations recorded at the 50 ppm doses (after 24 h) compared to the 25 ppm doses suggest that higher concentrations of monoterpenes (lime EOs and (*R*)-(+)-limonene) inhibit hyper-ammonia-producing bacteria, which promotes better nitrogen utilization by ruminants [44].

The present in vitro study indicates that higher doses (50 ppm) of (*R*)-(+)-limonene and lime essential oil, in particular, favorably modulate rumen fermentation toward a more propionogenic profile, improving the energetic efficiency of the system [45]. The reduction in CH_4_ in these variants was fully consistent with a significant increase in propionate concentration (*p* < 0.05) and a decrease in the acetate-to-propionate (A:P) ratio. This strongly suggests a shift in hydrogen metabolism—the excess hydrogen resulting from the destabilization of methanogen cell membranes [46] is redirected toward propionate synthesis at the expense of methanogenesis (the so-called hydrogen sink effect) [47].

Conversely, the divergent dynamics observed for the lower doses (25 ppm) of (*R*)-(+)-limonene—where the lowest CH_4_ production was recorded, but accompanied by a simultaneous decrease in propionate concentration and an increase in the A:P ratio—indicate that, at lower concentrations, methane mitigation processes rely on alternative, direct inhibitory mechanisms or other hydrogen utilization pathways, highlighting the complex, dose-dependent impact of monoterpenes on the rumen microbiome [48].

The reduction in CH_4_ observed in the limonene-treated variants was accompanied by an increase in propionate and a decrease in the acetate-to-propionate (A:P) ratio, suggesting a shift in hydrogen metabolism toward propionate synthesis at the expense of methanogenesis.

The bacterial community structure remained stable over the 14-day period, with no evident changes in diversity or composition between treatment and control groups. The community was dominated by rumen-characteristic Bacteroidota and Bacillota, whose relative abundances did not change significantly. While no global restructuring of the microbiome was observed, a decrease in the relative abundance of Methanobacteriota was detected in the (*R*)-(+)-limonene treatment. This may indicate a selective effect on methanogenic archaea. Nevertheless, the magnitude of this change was limited and warrants further functional validation.

The significant increase (*p* < 0.05) in glucose concentration observed in the limonene-treated group, accompanied by a decrease in NEFA levels, indicates a clear modulation of energy metabolism in cows. This metabolic profile suggests a reduced mobilization of fat reserves and a shift in energy balance toward a more efficient utilization of glucose as an energy substrate [49]. Similar relationships between glucose and NEFA have been reported in studies involving essential oil supplementation in dairy cows, where EOs improved energy utilization and reduced markers of lipolysis [50].

Essential oils may influence energy metabolism by modulating rumen fermentation and the availability of gluconeogenic precursors, particularly propionate [51], with both in vitro and in vivo results obtained in the present study supporting this mechanism. Consequently, the stabilization of metabolic parameters and reduced NEFA mobilization may occur, especially during periods of increased energy demand [52].

The absence of significant changes in liver enzyme activities (AST, ALT, and GGT) suggests that neither limonene nor its (*R*)-(+)-enantiomer exerts hepatotoxic effects. The stability of these parameters indicates no hepatocellular damage or metabolic overload of the liver, which is consistent with previous studies on essential oil supplementation in dairy cows. These studies have shown that EOs not only do not impair liver function but may also modulate nitrogen and energy metabolism [14,51,53,54]. This effect is associated with the influence of EOs on ion transport, nutrient absorption in the gastrointestinal tract, and modulation of rumen microbiota activity, among other processes [53]. Moreover, EO may positively affect cow health during late pregnancy, a period characterized by reduced immune responsiveness [55]. Additionally, the stable lipid profile (total cholesterol, HDL, LDL, and triglycerides) indicates that limonene supplementation does not disrupt lipid homeostasis. These findings are consistent with meta-analyses conducted in both beef and dairy cattle, which report no significant effect of EOs on blood lipid profiles [51].

Monitored rumen pH is a key indicator of fermentation stability [56]. During the initial phase of the experiment (days 0–7), no significant differences in rumen pH were observed, suggesting the absence of an immediate disruptive effect of supplementation on the acid–base balance. However, a significant increase in rumen pH was noted in the lime EO group on day 14 (6.96 vs. 6.66 in the control; *p* < 0.05), which may indicate a more pronounced effect on the rumen microbiome over time. At the same time, the lime EO group exhibited a stronger increase in HCO_3_^−^ and base excess (BE) compared to the (*R*)-(+)-limonene group, suggesting that compounds other than limonene contribute to the overall biological effect of the essential oil. The observed increase in rumen pH, together with elevated VFA concentrations in rumen fluid and lactate (LA) in blood, may indicate a selective modulation of the microbiome, including lactate-producing and/or lactate-utilizing bacteria [57,58]. Blood gas analysis indicates that supplementation with lime EO and (*R*)-(+)-limonene only moderately influenced the dynamics of acid–base parameters, which remained within physiological reference ranges, suggesting that the observed changes did not disrupt systemic homeostasis.

These mechanisms are consistent with the broader biological activity of essential oils, including their neuropharmacological and physiological effects [59,60]. The ability of (*R*)-(+)-limonene to readily cross biological barriers may explain its influence on the central regulation of respiratory homeostasis [61]. Monoterpenes, including limonene, have been shown to exert modulatory effects on the central nervous system, primarily via interaction with neurotransmitter systems such as GABAergic pathways, which may indirectly influence respiratory center activity [62]. The stability of blood pH observed in the present study, despite changes in pCO_2_ and HCO_3_^−^, suggests the presence of efficient compensatory mechanisms. Overall, the maintenance of acid–base balance and stable biochemical parameters confirms that the organism effectively buffers the metabolic effects of administered terpenoid compounds. Importantly, the lack of adverse effects on biochemical and acid–base parameters supports the safety of limonene and related terpenes as dietary additives.

Overall, the present findings indicate that lime essential oil exerted a broader and more complex biological effect than pure (*R*)-(+)-limonene, suggesting that interactions among multiple bioactive constituents contribute to its activity. The observed responses were dose-dependent, particularly under in vitro conditions, where higher inclusion levels more effectively modulated rumen fermentation toward reduced methane production and enhanced propionate formation. Importantly, these effects occurred without adverse alterations in biochemical, hepatic, or acid–base parameters, confirming the maintenance of systemic homeostasis. Collectively, the results support the potential application of lime essential oil and limonene-based preparations as natural feed additives capable of improving rumen fermentation efficiency while maintaining animal safety.

A limitation of the present study is the relatively small number of animals and the use of non-lactating dairy cows. Although the crossover design with repeated measures and linear mixed-effect modeling helped to increase statistical power, the possibility of type II errors cannot be excluded for variables with high biological variability, such as methane concentration. Furthermore, while non-lactating, rumen-cannulated cows represent a standard and widely used model that enables repeated sampling under controlled conditions and minimizes confounding from lactation physiology, the results may not be directly transferable to high-producing lactating cows, in which higher dry matter intake, faster rumen passage rates, altered energy metabolism and different microbial activity could modify the response to lime essential oil and (*R*)-(+)-limonene. Additionally, the experimental period was relatively short and functional analyses of the rumen microbiome were not performed, which limits the interpretation of the long-term biological effects and microbial mechanisms associated with supplementation. Therefore, confirmation of the observed effects in lactating dairy cows under commercial conditions, together with longer-term studies including functional microbiome analyses, is warranted before practical recommendations can be made.

## 4. Materials and Methods

### 4.1. Essential Oils and Chemicals

The essential oils analyzed in the present study were obtained from a commercial supplier with established market credibility, namely Avicenna Oil (Wrocław, Poland). The supplier was selected based on prior experience and the consistent quality of the essential oils provided, ensuring the reliability and reproducibility of the experimental material. The tested essential oils included those derived from *Citrus sinensis*, *Citrus limon*, *Citrus aurantifolia*, *Cymbopogon citratus*, *Rosmarinus officinalis*, and *Mentha piperita*. The (*R*)-(+)-limonene (≥98% purity) used in this study was purchased from Sigma-Aldrich (Darmstadt, Germany).

### 4.2. Chromatographic Analysis of Essential Oils

Prior to chromatographic analysis, essential oils were diluted by mixing 20 µL of the sample with 980 µL of dichloromethane (GC–MS grade; Sigma-Aldrich, Darmstadt, Germany). The resulting solution was then transferred into a chromatographic vial.

The chemical composition of the oil was determined using gas chromatography–mass spectrometry (GC–MS). Analyses were performed on Shimadzu GC–MS QP 2020 (Shimadzu, Kyoto, Japan), and the separation of volatile constituents was achieved using a Zebron ZB-5 MSi capillary column (30 m × 0.25 mm × 0.25 µm; Phenomenex, Torrance, CA, USA). Helium was used as the carrier gas at a constant flow rate of 1.08 mL min^−1^, with a split ratio of 1:49. Mass spectra were recorded over a range of 50–350 m/z at a scan rate of 0.3 scans s^−1^. The oven temperature program started at 70 °C, followed by an increase to 200 °C at 5 °C min^−1^, and then to 280 °C at 20 °C min^−1^. The final temperature was maintained for 3 min, resulting in a total run time of 34 min. A 1 µL aliquot of the prepared solution was injected at 260 °C.

To confirm the enantiomeric purity of (*R*)-(+)-limonene, an additional analysis was performed using a chiral capillary column (Cydex-B, 50 m × 0.25 mm × 0.25 µm; Trajan Scientific Europe Ltd., Milton Keynes, UK) on the same GC–MS system. The injector temperature was set at 220 °C. The oven temperature program was initiated at 60 °C and held for 2 min, followed by a gradual increase to 150 °C at a rate of 3 °C min^−1^, and then raised to 220 °C at 25 °C min^−1^. Helium was used as the carrier gas at a constant flow rate of 0.9 mL min^−1^, with a split ratio of 1:75. The MS conditions included an interface temperature of 210 °C and an ion source temperature of 250 °C. Mass spectra were recorded over a scan range of 35–350 *m*/*z*.

Identification of volatile compounds was performed using a multi-step approach. Mass spectra corresponding to individual peaks were compared with those available in the NIST 23 (National Institute of Standards and Technology) and FFNSC (Flavors and Fragrances of Natural and Synthetic Compounds) libraries. Data processing was conducted using AMDIS software (version 2.73) and GCMSsolution (version 4.20; Shimadzu, Kyoto, Japan). Additionally, linear retention indices (LRIs) were calculated and subsequently verified against reference values reported in the NIST 23 and FFNSC databases.

### 4.3. Cultures of Rumen Microorganisms—In Vitro Study

The effect of essential oils on the growth of selected ruminal bacterial strains was assessed using liquid Schaedler broth inoculated with bacterial cultures derived from single colonies previously grown on solid media. The bacterial strains used in this study were identified according to the methodology described in previous studies [63].

Following anaerobic incubation for 48 h at 39 °C, the resulting starter cultures were transferred into media containing a series of twofold dilutions of essential oils. The inoculum density was standardized to 0.5 McFarland units in accordance with CLSI guidelines [63].

Prior to application, essential oils were dissolved in dimethyl sulfoxide (DMSO) at a 1:1 ratio and added aseptically to the culture medium after sterilization to obtain final concentrations ranging from 25 to 6400 ppm. The assays were performed in 96-well microplates.

Cultures of *Lactobacillus delbrueckii* subsp. *lactis* and *Streptococcus bovis* were incubated under anaerobic conditions for 24 h at 39 °C, whereas *Prevotella albensis* and *Butyrivibrio fibrisolvens* were incubated for 48 h at the same temperature. Anaerobic conditions were ensured using a GasPak EZ anaerobe pouch system (BD).

Bacterial growth was monitored by measuring optical density at 650 nm using a Spark microplate reader (Tecan, Männedorf, Switzerland). All procedures were conducted in a Coy anaerobic chamber under strictly controlled conditions, with oxygen levels maintained below 0.5%.

Based on the obtained data, minimum inhibitory concentration (MIC) and half-maximal inhibitory concentration (IC_50_) values were determined. The percentage inhibition at each concentration of essential oil was calculated relative to the untreated control. MIC was defined as the lowest concentration resulting in at least 90% inhibition of bacterial growth, whereas IC_50_ corresponded to a 50% reduction in optical density compared to the control.

### 4.4. In Vitro Fermentation Study

In vitro experiments were conducted to evaluate the effects of lime essential oil and (*R*)-(+)-limonene on rumen fermentation characteristics and methane production. The results of the in vitro study were used to determine the appropriate dose of the additive for subsequent in vivo experiments. For these experiments, rumen fluid was obtained from three non-lactating Polish Holstein–Friesian cows (BW 600 ± 30 kg) fitted with permanent ruminal cannulas and maintained on the basal diet described in Section 4.5. Sampling was performed approximately 3 h after the morning feeding, corresponding to the postprandial peak of ruminal fermentative activity (Section 4.5). All experimental procedures involving animals were approved by the Local Ethics Committee on Animal Experiments (Protocol No. 058/2024).

In vitro fermentation was conducted using the Ankom RF gas production system (ANKOM Technology, Macedon, NY, USA). Prior to incubation, rumen fluid was thoroughly homogenized and combined with a buffer solution preheated to 39 °C at a ratio of 1:4 (75 mL rumen fluid to 300 mL buffer). The resulting inoculum was then distributed into 500 mL glass fermentation bottles that had been pre-equilibrated to incubation temperature. Each treatment was performed in triplicate in each incubation run, and the tested compounds were applied directly to the substrate immediately before incubation to ensure uniform distribution within the fermentation system. Each fermentation bottle contained Ankom filter bags filled with 1 g of feed substrate. In the experimental groups, additional bags supplemented with the tested essential oil were introduced. Before sealing, the inoculum was flushed with carbon dioxide to establish anaerobic conditions, and the bottles were subsequently placed in a shaking water bath maintained at 39 °C. Fermentation was carried out under strictly anaerobic conditions and constant temperature throughout the incubation period to ensure stable microbial activity.

The essential oil applied in the fermentation experiment was selected based on the results obtained from the in vitro assays of rumen microbial cultures described in Section 2.1. The experimental setup consisted of a control group without supplementation (CON) and treatment groups receiving the essential oil at two inclusion levels (25 and 50 ppm). These concentrations were chosen for further evaluation of their effects on rumen fermentation parameters.

### 4.5. In Vivo Supplementation Trial—Animals, Design, and Treatments

The objective of the in vivo experiment was to evaluate the effects of dietary supplementation with lime essential oil and (*R*)-(+)-limonene on rumen fermentation, methane production, microbiota composition, and metabolic parameters in dairy cows. Rumen fluid was obtained from non-lactating Polish Holstein–Friesian cows fitted with ruminal cannulas, with an average body weight of 600 ± 30 kg. Based on NRC/NASEM [64] tabular values for feed ingredients, the estimated average nutritive value of the basal diet was approximately as follows: crude protein 20.3% DM, NDF 46.7% DM, ADF 30.3% DM, and NEL 5.9 MJ kg^−1^ DM. These values should be interpreted as estimated tabular values rather than analytically determined nutrient concentrations. The composition and nutritive value of the basal diet were kept constant across all experimental groups throughout the study period. Throughout the experiment, all animals had unrestricted (ad libitum) access to fresh drinking water. All experimental procedures involving animals were approved by the Local Ethics Committee on Animal Experiments (Protocol No. 058/2024). Using a crossover design, cows were randomly assigned to one of three treatment groups (n = 4 per group): control (CON), lime EO, and (*R*)-(+)-limonene. Animals received 2.5 mL day^−1^ of either lime essential oil or (*R*)-(+)-limonene administered directly into the rumen for 14 consecutive days. The animals received 2.5 mL/head/day of essential oil via a rumen cannula. The dosage was selected based on preliminary in vitro experiments identifying biologically effective concentrations.

Rumen fluid and blood samples were collected approximately 3 h after the morning feeding to standardize sampling conditions and to capture the postprandial period characterized by active ruminal fermentation and nutrient absorption. This interval is commonly used in ruminant studies because it reflects dynamic changes in ruminal fermentation end-products, including volatile fatty acids and ammonia nitrogen, as well as associated short-term metabolic responses in blood. Sampling at a fixed post-feeding interval minimized the variation associated with feeding behavior and ensured comparability among animals, treatments, and sampling days [65]. During each collection, about 3 L of rumen fluid was obtained. Immediately after sampling, the material was transferred into pre-warmed insulated containers to preserve physiological temperature (39 °C). Before use in further analyses, the rumen fluid was filtered through three layers of cheesecloth to remove large particulate matter. Ruminal fluid was sampled on days 0, 3, 7, and 14 of the experimental period for analysis of pH, NH_3_-N, NH_3_, NH_4_^+^, and VFA. Blood and rumen gas samples for biochemistry, acid–base balance, and CH_4_ analysis were collected on days 0, 7, and 14. Samples for microbial 16S rRNA gene sequencing were taken on day 0 (T0) and at 14 days (T14).

Blood samples were collected into sterile serum tubes (Sarstedt, Nümbrecht, Germany). To obtain serum, samples were centrifuged within 2 h of collection at 3000× *g* for 10 min at room temperature and subsequently stored at −20 °C until analysis. Biochemical parameters were determined using a Pentra 400 analyzer (Horiba ABX Diagnostics, Grabels, France). Glucose concentration was measured using the glucose oxidase method. Concentrations of non-esterified fatty acids (NEFAs) were assessed enzymatically with Randox reagents (Crumlin, Dublin, Ireland), whereas triglycerides (TGs) and total cholesterol were determined using enzymatic assays (Horiba ABX, Montpellier, France). High-density lipoprotein (HDL) and low-density lipoprotein (LDL) cholesterol levels were quantified through colorimetric methods.

Liver function markers, including aspartate aminotransferase (AST) and gamma-glutamyl transferase (GGT), were analyzed using kinetic assays. Total protein (TP) and albumin (Alb.) concentrations were determined colorimetrically using commercial reagents (Horiba ABX, Montpellier, France).

The antioxidant status was also evaluated. Total antioxidant capacity (TAS) in serum was determined using a colorimetric assay based on the ABTS method. Glutathione reductase (GR) activity in whole blood was measured enzymatically by monitoring the oxidation of NADPH to NADP^+^ at 340 nm. All measurements were carried out using a Synergy multi-mode microplate reader equipped for fluorescence, luminescence, and absorbance detection (BioTek Instruments, Winooski, VT, USA).

Next, 2 mL blood samples were collected from the coccygeal vein using heparinized plastic syringes (Sarstedt, Poland). Immediately after sampling, any air bubbles were carefully removed, and the syringe was sealed with a rubber cap to prevent gas exchange. Blood pH, partial pressures of oxygen (pO_2_) and carbon dioxide (pCO_2_), bicarbonate concentration (HCO_3_^−^), and base excess (BE) were determined using an Edan i15 blood gas analyzer (Edan Instruments, Shenzhen, China).

### 4.6. Sample Collection and Ammonia Analysis

After 4 and 24 h of incubation, samples of fermentation fluid were collected and placed into 15 mL Falcon tubes (10 mL per sample). To terminate microbial activity and preserve volatile components, 0.5 mL of concentrated sulfuric acid (H_2_SO_4_) was added immediately to each sample. The two-timepoint design therefore allows for discrimination between immediate and prolonged effects of the tested additives on rumen fermentation parameters and methane production.

Before analysis, a 1 mL aliquot of the treated sample was centrifuged at 14,500× *g* for 4 min to remove insoluble material. The supernatant was then diluted with deionized water (1:9, *v*/*v*) to adjust ammonia levels to the measurable range of the instrument.

Ammonia concentration was measured using a portable colorimeter (Hanna Instruments, Smithfield, RI, USA; model HI-97733) with appropriate reagents, in accordance with the Nessler method [66] and the manufacturer’s protocol. Final values were obtained directly from the instrument based on its internal calibration. Similar methods were used in the in vivo experiment (Section 4.5).

### 4.7. Volatile Fatty Acid Analysis

For volatile fatty acid (VFA) determination, 1 mL samples obtained from the incubation experiment were transferred into 2 mL Eppendorf tubes. Heptanoic acid (C_7_), used as an internal standard, was added to each sample at a final amount of 0.5 mg. Subsequently, 0.8 mL of diethyl ether was added, and the samples were mixed thoroughly using a vortex mixer for 30 s, followed by centrifugation at 14,000× *g* for 4 min to achieve phase separation.

After centrifugation, the organic phase was collected and purified by filtration through a celite layer. The aqueous phase was then subjected to a second extraction with an additional 0.8 mL of diethyl ether. Following repeated vortexing (30 s) and centrifugation under the same conditions, the obtained extract was filtered as described above. The organic fractions from both extraction steps were combined, transferred into chromatographic vials, and subjected to GC-MS analysis.

VFA profiling was carried out using a Shimadzu GC–MS QP 2020 system (Shimadzu, Kyoto, Japan), the same instrument employed for essential oil characterization. Separation was performed on a Zebron ZB-FAME capillary column (60 m × 0.25 mm × 0.20 μm; Phenomenex, Torrance, CA, USA). The oven temperature program was initiated at 80 °C and held for 1 min, followed by a ramp to 200 °C at 7 °C min^−1^ and a final hold of 2 min. Mass spectra were recorded over a range of 42–300 *m*/*z* at a scan rate of 1 scan s^−1^. Helium was used as the carrier gas at a flow rate of 1.80 mL min^−1^. The injector temperature was set at 260 °C, and the split ratio was 50. Quantification of individual VFAs was performed using calibration curves constructed from authentic reference standards. The methodology applied in this study is described in Section 4.4 and Section 4.5.

### 4.8. Methane Analysis

Methane (CH_4_) concentrations were quantified using gas chromatography. Measurements were carried out on a Shimadzu GC-2030 system (Shimadzu Corporation, Kyoto, Japan) equipped with a flame ionization detector (FID) and an SH-Q-BOND capillary column (30 m × 0.32 mm × 10 µm). The injector was maintained at 240 °C and operated in split mode (1:10). Helium served as the carrier gas at a linear velocity of 35.0 cm s^−1^.

The oven temperature was initially set to 40 °C and held for 2.5 min, followed by a temperature ramp to 200 °C at a rate of 35 °C min^−1^. The final temperature was maintained for 2.0 min, resulting in a total analysis time of 9.07 min. The FID temperature was kept at 240 °C, with gas flows set to 32.0 mL min^−1^ for hydrogen, 200.0 mL min^−1^ for air, and 24.0 mL min^−1^ for helium as the makeup gas.

Methane production under in vitro conditions was assessed during fermentation experiments. Gas samples were withdrawn from the headspace of incubation vessels after 4 and 24 h using gas-tight Hamilton syringes (600 µL) and injected directly into the chromatograph.

For in vivo measurements, gas samples were collected into 1 L Tedlar^®^ bags (Sigma-Aldrich, St. Louis, MO, USA) using a sterile sampling setup. Gas was drawn at a constant flow rate of 2 L min^−1^ with a calibrated GilAir-3 EX/ATEX pump (Sensidyne, St. Petersburg, FL, USA). Prior to sampling, the rumen cannula opening was sealed to prevent contamination with ambient air and to preserve anaerobic conditions. The sampling system was flushed before each collection to avoid cross-contamination.

Gas was collected until the desired volume of 1 L was reached, after which the bags were immediately sealed, labeled, and transported to the laboratory under conditions protecting them from direct light exposure. Analyses were performed promptly after collection. Gas samples were introduced directly from the Tedlar^®^ bags into the GC system via a gas-tight interface connected to the sampling loop. Measurements were conducted on days 0, 3, 7, and 14 under standardized conditions. The methodology applied in this study is described in Section 4.4 and Section 4.5.

### 4.9. Microbial Community Analysis

Four experimental groups were defined to evaluate the effects of pine essential oil supplementation in dairy cows over a 14-day in vivo experiment: CTRL_T0 and CTRL_T14 represent the control cows at baseline and after 14 days, and PINE_EO_T0 and PINE_EO_T14 represent the treated cows before and after supplementation, respectively (Section 4.5). The control group included six biological replicates per timepoint, whereas the treatment groups comprised four biological replicates each.

#### 4.9.1. DNA Extraction and Purification

Rumen fluid samples were collected and filtered through sterile gauze to remove large feed particles, then immediately stored at −80 °C until further processing. Genomic DNA was extracted using the MasterPure DNA Purification Kit (LGC Genomics, Teddington, UK) according to the manufacturer’s protocol. An additional clean-up step was performed using the GeneMATRIX Stool DNA Purification Kit (EURx, Gdańsk, Poland) to improve DNA purity.

#### 4.9.2. Library Preparation and Sequencing

Amplification of the V3–V4 region of the bacterial 16S rRNA gene was performed using a two-step PCR approach with primers 341F (5′-CCTACGGGNGGCWGCAG-3′) and 785R (5′-GACTACHVGGGTATCTAATCC-3′). The first PCR generated target amplicons, while the second step incorporated dual indices and Illumina-compatible sequencing adapters. Following each PCR step, amplicons were purified using magnetic bead-based clean-up. DNA concentration was quantified fluorometrically using PicoGreen (Thermo Fisher Scientific, Waltham, MA, USA). Paired-end sequencing was carried out on the Aviti platform (Element Biosciences, San Diego, CA, USA) at Genomed S.A. (Warsaw, Poland).

#### 4.9.3. Bioinformatics Processing

Raw reads were demultiplexed and converted to FASTQ format using vendor-provided software. Downstream processing was conducted in QIIME2 (version 2024.5) [67] using the SILVA 138.2 reference database [68]. Adapter and primer sequences were removed using Cutadapt (version 4.7) [69], followed by quality filtering based on a minimum read length of 30 bp. Denoising, paired-end read merging, and chimera removal were performed using the DADA2 plugin [70], resulting in Amplicon Sequence Variants (ASVs). Taxonomic assignment was performed using a hybrid approach against the SILVA database. Initial classification was conducted using VSEARCH [71] based on sequence similarity (minimum identity 50% and coverage 80%), followed by refinement of unclassified sequences using a Naive Bayes classifier with a confidence threshold of 0.7.

Alpha diversity was assessed using Observed ASVs, Chao1, Shannon, Simpson, and Inverse Simpson indices to capture both richness and evenness of microbial communities. Beta diversity was evaluated using Bray–Curtis dissimilarity matrices and visualized through Principal Coordinate Analysis (PCoA).

### 4.10. Statistical Analysis

All statistical procedures were conducted using the Statistica 13.3 software package (TIBCO Software Inc., Palo Alto, CA, USA). Prior to data analysis, the assumptions underlying the applied statistical models were verified. The normality of variable distributions and model residuals was evaluated using the Shapiro–Wilk test. For the in vitro experiment, where all observations were independent, the data were analyzed using a two-way analysis of variance (ANOVA). When significant differences were detected, multiple comparisons among means were performed using Tukey’s post hoc procedure. Due to the repeated-measures nature of the in vivo experiment, including repeated sampling of the same animals at different timepoints, blood biochemical variables and rumen fermentation parameters were analyzed using a linear mixed-effect model. In this approach, dietary supplementation (group: control vs. experimental group), sampling time (0, 7, and 14 days), and their interaction (group × time) were treated as fixed effects, whereas animal identity was incorporated as a random effect to account for repeated observations and individual variability among animals. Whenever statistically significant effects were identified, pairwise comparisons were additionally carried out using Tukey’s HSD test. Differences in microbial composition between experimental groups were further examined by permutational multivariate analysis of variance (PERMANOVA) calculated using Bray–Curtis distance matrices. The obtained results are expressed as mean values ± standard error of the mean (SEM). Statistical significance was established at *p* ≤ 0.05, while values within the range of 0.05 < *p* ≤ 0.10 were considered indicative of a statistical tendency.

## 5. Conclusions

GC–MS analysis demonstrated that lime essential oil (*Citrus aurantifolia*) was composed predominantly of monoterpene hydrocarbons, with limonene identified as the principal constituent. Both lime essential oil and (*R*)-(+)-limonene exhibited antimicrobial activity under in vitro conditions: both additives modulated rumen fermentation in a dose-dependent manner, with 50 ppm identified as the most effective supplementation level. In addition, methane production was reduced compared with the control treatment. In the in vivo study, lime essential oil exerted a more consistent effect on rumen function than isolated limonene, as indicated by an increased ruminal pH and total volatile fatty acid concentration after 14 days of supplementation (*p* < 0.05). Supplementation was well tolerated and did not adversely affect the major biochemical indicators of animal health, and only moderate changes in glucose concentration and selected acid–base balance parameters were observed. Microbial community analysis indicated the overall stability of the rumen microbiota, although (*R*)-(+)-limonene reduced the relative abundance of Methanobacteriota. Overall, our findings suggest that lime essential oil may represent a promising natural feed additive for modulating rumen fermentation and metabolic responses in dairy cows. Further studies are required to evaluate its long-term effects under practical production conditions.

## Figures and Tables

**Table 1:** MIC and IC50 values for selected limonene-rich plant materials and limonene enantiomers against four bacterial strains. All data points represent the mean values from three independent replicates (n = 3).

Bacterial Strain	Essential Oil or Pure Compound	MIC ^1^ (ppm)	IC_50_ ^2^ (ppm)	GS ^3^ (ppm)	
*Butyrivibrio fibrisolvens*	*Citrus sinensis*	*1600*	800	200	1.6×
	*Citrus limon*	*1600*	400	-	-
	*Citrus aurantifolia*	*800*	200	25	1.1×
	*Cymbopogon citratus*	*200*	50	-	-
	*Rosmarinus officinalis*	1600	400	-	-
	*Mentha piperita*	1600	200	-	-
	(*S*)-(−)-Limonene	3200	400	-	-
	(*R*)-(+)-Limonene	4000	100	-	-
*Prevotella albensis*	*Citrus sinensis*	1600	400	25	2×
	*Citrus limon*	1600	400	50	1.3×
	*Citrus aurantifolia*	200	100	50	1.4×
	*Cymbopogon citratus*	200	50	-	-
	*Rosmarinus officinalis*	1600	800	-	-
	*Mentha piperita*	800	200	-	-
	(*S*)-(−)-Limonene	400	25	-	-
	(*R*)-(+)-Limonene	1600	100	-	-
*Lactobacillus delbrueckii*	*Citrus sinensis*	1600	200	-	-
	*Citrus limon*	400	200	-	-
	*Citrus aurantifolia*	200	100	-	-
	*Cymbopogon citratus*	1600	400	-	-
	*Rosmarinus officinalis*	800	600	-	-
	*Mentha piperita*	400	100	-	-
	(*S*)-(−)-Limonene	6400	400	-	-
	(*R*)-(+)-Limonene	1600	1200	-	-
*Streptococcus bovis*	*Citrus sinensis*	3200	1600	-	-
	*Citrus limon*	NT	NT	-	-
	*Citrus aurantifolia*	1600	800	-	-
	*Cymbopogon citratus*	800	400	-	-
	*Rosmarinus officinalis*	3200	800	-	-
	*Mentha piperita*	3200	1600	200	1.3×
	(*S*)-(−)-Limonene	6400	3200	-	-
	(*R*)-(+)-Limonene	6400	3200	-	-

^1^ Minimum inhibitory concentration; ^2^ half maximal inhibitory concentration; ^3^ growth stimulation is expressed as fold increase relative to control; NT—not detected.

**Table 2:** Chemical composition of lime essential oil determined using GC-MS analysis.

No	Compound	RI_exp_ ^1^	RI_lit_ ^2^	Area ^3^ (%)	Ident. ^4^
1	α-Pinene	942	933	0.63	RI, MS, S
2	Camphene	949	953	2.55	RI, MS, S
3	α-Fenchene	950	954	0.08	RI, MS, S
4	Sabinene	980	972	2.35	RI, MS, S
5	β-Pinene	986	978	12.01	RI, MS, S
6	Myrcene	991	991	1.29	RI, MS, S
7	δ-3-Carene	1012	1009	0.09	RI, MS, S
8	α-Terpinene	1019	1018	0.05	RI, MS, S
9	*p*-Cymene	1026	1025	6.50	RI, MS, S
10	Limonene	1033	1030	46.57	RI, MS, S
11	*trans*-β-Ocimene	1045	1046	0.12	RI, MS, S
12	γ-Terpinene	1059	1058	10.71	RI, MS, S
13	*cis*-Sabinene hydrate	1071	1069	0.07	RI, MS, S
14	Terpinolene	1087	1086	0.42	RI, MS, S
15	Linalool	1099	1101	0.22	RI, MS, S
16	*trans*-Sabinene hydrate	1103	1102	0.07	RI, MS, S
17	*trans*-*p*-Mentha-2,8-dien-1-ol	1122	1122	0.05	RI, MS, S
18	*cis*-Limonene oxide	1134	1134	0.32	RI, MS
19	*trans*-Limonene oxide	1138	1138	0.35	RI, MS
20	*trans*-Myroxide	1444	1441	0.35	RI, MS, S
21	Citronellal	1150	1152	0.04	RI, MS,
22	Isocyclocitral	1163	1156	0.13	RI, MS
23	Neomenthol	1168	1170	0.11	RI, MS
24	Borneol	1174	1173	0.03	RI, MS, S
25	Lavandulol	1180	1182	0.05	RI, MS, S
26	Terpinen-4-ol	1183	1184	0.13	RI, MS, S
27	α-Terpineol	1196	1195	0.43	RI, MS, S
28	*p*-Menth-3-en-7-al	1199	1196	0.12	RI, MS
29	*cis*-4-Caranone	1202	1205	0.11	RI, MS
30	Decanal	1204	1208	0.03	RI, MS, S
31	*trans*-Carveol	1219	1223	0.16	RI, MS
32	Nerol	1224	1229	0.11	RI, MS, S
33	7-Octen-1-ol, 3,7-dimethyl-	1229	1230	0.14	RI, MS
34	*cis*-Carveol	1232	1232	0.05	RI, MS, S
35	Neral	1237	1238	1.08	RI, MS, S
36	Carvone	1245	1246	0.04	RI, MS, S
37	Geranial	1266	1268	1.73	RI, MS, S
38	Lavandulyl acetate	1275	1284	0.06	RI, MS, S
39	Limonen-10-ol	1281	1290	0.04	RI, MS
40	Bornyl acetate	1285	1285	0.01	RI, MS
41	δ-Elemene	1338	1335	0.06	RI, MS
42	Citronellyl acetate	1344	1350	0.31	RI, MS
43	*cis*-Geranyl acetate	1356	1361	1.75	RI, MS
44	*trans*-Geranyl acetate	1375	1380	0.34	RI, MS
45	β-Elemene	1393	1390	0.13	RI, MS
46	*cis*-α-Bergamotene	1416	1416	0.13	RI, MS
47	α-Santalene	1422	1418	0.03	RI, MS, S
48	*trans*-Caryophyllene	1425	1425	0.37	RI, MS, S
49	*trans*-α-Bergamotene	1436	1432	1.86	RI, MS, S
50	Aromadendrene	1439	1438	0.10	RI, MS
51	*trans*-β-Farnesene	1452	1452	0.12	RI, MS
52	β-Santalene	1459	1462	0.11	RI, MS
53	*trans*-β-Bergamotene	1487	1483	0.11	RI, MS
54	β-Selinene	1494	1492	0.03	RI, MS
55	*trans*-α-Bisabolene	1501	1503	0.32	RI, MS
56	*trans*,*trans*-α-Farnesene	1504	1504	0.29	RI, MS
57	β-Bisabolene	1510	1508	2.75	RI, MS, S
58	γ-Cuprenene	1522	1530	0.01	RI, MS, S
59	*trans*-α-Bisabolene	1542	1540	0.06	RI, MS
60	*cis*-Sesquisabinene hydrate	1545	1544	0.07	RI, MS
61	Caryophyllene oxide	1587	1588	0.92	RI, MS, S
62	Humulene epoxide II	1615	1613	0.08	RI, MS
63	1,10-di-epi-Cubenol	1620	1614	0.12	RI, MS
64	Muurola-4,10(14)-dien-1-beta-ol	1632	1632	0.07	RI, MS
65	*cis*-Nerolidyl acetate	1659	1665	0.10	RI, MS
66	α-Bisabolol	1689	1688	0.03	RI, MS
67	14-hydroxy-4,5-dihydro-Caryophyllene	1705	1712	0.11	RI, MS
68	Hernianin	1720	1730	0.13	RI, MS
69	8-α-Acetoxyelemol	1785	1793	0.03	RI, MS
70	*cis*-11-Hexadecenal	1817	1804	0.07	RI, MS

^1^ Experimentally calculated linear retention index; ^2^ literature-derived retention index according to the NIST 23 Mass Spectral Library; ^3^ calculated by peak area normalization, ^4^ identification method: RI (retention index), MS (mass spectra, NIST23 database), S (standard of compound).

**Table 3:** Effects of lime essential oil and (*R*)-(+)-limonene on in vitro fermentation characteristics.

Time (Hours)	CON	Lime EO		(*R*)-(+)-Limonene		SEM	*p*-Value
		25 ppm	50 ppm	25 ppm	50 ppm		
pH							
4 h	6.64	6.76	6.75	6.67	6.73	0.02	0.06
24 h	6.55	6.69	6.57	6.70	6.80	0.05	0.03
NH_3_-N, mg L^−1^							
4 h	175.43	202.34	176.34	213.43	198.67		0.12
24 h	198.2	223.60	182.50	232.00	219.53	9.08	0.18
NH_3_, mg L^−1^							
4 h	123.34	234.24	198.34	234.67	243		0.09
24 h	241.13	272.00	217.50	282.00	267.07	11.74	0.02
NH_4_^+^, mg L^−1^							
4 h	205.13	190.89	214.22	265,71	243,12		0.07
24 h	230.23	216.00	238.00	299.00	282.73	15.99	0.02
Total VFA, mg mL^−1^							
4 h	1.40	1.59	1.22	0.97	1.46	0.11	0.06
24 h	1.29	1.54	1.17	0.87	1.51	0.12	0.07
Individual VFA mg mL^−1^							
Acetate							
4 h	0.747	0.873	0.576	0.594	0.810	0.06	0.09
24 h	0.656	0.529	0.790	0.506	0.788	0.06	0.16
Propionate							
4 h	0.365	0.377	0.365	0.190	0.351	0.04	0.11
24 h	0.365 ^a^	0.360 ^a^	0.430 ^b^	0.187 ^a^	0.429 ^b^	0.05	0.14
Butyrate							
4 h	0.291	0.345	0.283	0.187	0.297	0.03	0.23
24 h	0.271	0.311	0.287	0.182	0.295	0.02	0.07
Acetate to propionate ratio							
4 h	2.05	2.32	1.58	3.13	2.31	0.25	0.17
24 h	1.80	1.47	1.84	2.71	1.84	0.21	0.09
Methane, mL L^–1^							
4 h	14.06	16.24	19.83	16.80	14.52	2.78	0.13
24 h	58.34	48.72	48.20	45.41	49.45	3.21	0.08

^a,b^—Means with different superscript letters differ significantly between groups (*p* < 0.05).

**Table 4:** Ammonia (NH_3_), NH_3_-N, NH_4_^+^, volatile fatty acids (VFAs), and ruminal pH in cows supplemented with lime essential oil and (*R*)-(+)-limonene.

Time (Days)	CON	Lime EO	(*R*)-(+)-Limonene	SEM	*p*-Value		
					TG	T	TG × T
pH							
0	6.82	6.75	6.91	0.08	0.55	<0.01	0.11
3	6.71	6.51	6.45	0.17			
7	6.61	6.62	6.71	0.10			
14	6.66 ^b^	6.96 ^a^	6.54 ^b^	0.14			
NH_3_-N, mg L^−1^							
0	143.5	130.3	104.3	43.1	0.948	0.01	0.61
3	136.0 ^ab^	113.5 ^b^	197.8 ^a^	63.3			
7	192.8	116.0	181.0	50.8			
14	124.0	117.3	109.8	61.5			
NH_3_, mg L^−1^							
0	181.3	158.5	129.8	60.1	0.55	0.02	0.55
3	166.5 ^ab^	173.5 ^b^	142.3 ^a^	76.8			
7	235.3	141.4	198.5	62.0			
14	149.3	146.5	134.8	75.3			
NH_4_^+^, mg L^−1^							
0	178.0	149.4	131.3	53.44	0.63	0.01	0.42
3	175.3 ^ab^	139.3 ^b^	152.5 ^a^	78.49			
7	165.8	149.6	174.3	62.99			
14	159.0	148.3	164.5	76.26			
Total VFA, mg mL^−1^							
0	3.78	3.21	4.02	0.25	0.95	0.05	0.26
3	2.89 ^ab^	2.42 ^b^	3.96 ^a^	1.25			
7	4.03	3.38	4.31	0.40			
14	4.11 ^b^	4.84 ^a^	4.21 ^b^	1.10			
Individual VFA, mg mL^−1^							
Acetate							
0	2.57	2.14	2.60	0.18	0.98	0.04	0.17
3	1.81 ^ab^	1.54 ^b^	2.27 ^a^	0.75			
7	2.61	2.25	2.77	0.25			
14	2.68 ^b^	3.38 ^a^	2.69 ^b^	0.80			
Propionate							
0	0.71	0.61	0.78	0.05	0.91	0.12	0.23
3	0.58 ^ab^	0.46 ^b^	0.76 ^a^	0.20			
7	0.86	0.69	0.88	0.10			
14	0.71	0.85	0.89	0.23			
Butyrate							
0	0.50	0.46	0.63	0.04	0.82	0.11	0.24
3	0.50 ^ab^	0.42 ^b^	0.93 ^a^	0.15			
7	0.56	0.45	0.66	0.05			
14	0.62	0.75	0.63	0.08			
Acetate to propionate ratio							
0	3.62	3.51	3.33	0.14	0.34	0.09	0.27
3	3.12	3.35	2.99	0.18			
7	3.04	3.26	3.15	0.11			
14	3.15	4.76	3.02	0.97			
Methane, ppm							
0	7202.8	6812.4	7794.0	1580.4	0.44	0.23	0.31
3	8565.3	10,995.8	6586.5	4500.2			
7	7992.2	5966.3	5298.0	3655.4			
14	7981.0	6755.7	7647.4	7450.8			

*p*-Value: TG for the effect of group, T for the effect of time, and TG × T for the interaction between group and time; ^a,b^—Means with different superscript letters differ significantly between groups (*p* < 0.05).

**Table 5:** Effect of dietary supplementation with lime essential oil and (*R*)-(+)-limonene on selected serum biochemical parameters.

Parameter	Time (Days)	CON	Lime EO	(*R*)-(+)-Limonene	SEM	*p*-Value		
						TG	T	TG × T
Glucose (mmol L^−1^)	0	3.48	3.85	3.58	0.15	0.12	0.02	<0.01
	7	3.73	3.77	3.86	0.06			
	14	3.63 ^a^	3.92 ^b^	3.38 ^a^	0.12			
NEFA ^8^ (mmol L^−1^)	0	0.21	0.18	0.25	0.01	0.33	0.05	0.13
	7	0.26	0.24	0.25	0.02			
	14	0.30	0.24	0.32	0.03			
Chol ^1^ (mmol L^−1^)	0	2.27	2.12	2.41	0.13	0.34	0.16	0.42
	7	2.38	2.47	2.44	0.11			
	14	2.06	2.20	2.28	0.09			
HDL ^4^ (mmol L^−1^)	0	1.49	1.30	1.49	0.06	0.31	0.19	0.42
	7	1.49	1.47	1.59	0.05			
	14	1.37	1.38	1.44	0.06			
LDL ^5^ (mmol L^−1^)	0	0.25	0.23	0.28	0.02	0.44	0.22	0.51
	7	0.27	0.30	0.26	0.02			
	14	0.23	0.24	0.26	0.02			
TG ^6^ (mmol L^−1^)	0	0.15	0.16	0.19	0.01	0.81	0.11	0.68
	7	0.19	0.18	0.18	0.01			
	14	0.18	0.17	0.18	0.01			
Creatinine (µmol L^−1^)	0	87.33	89.10	95.98	2.94	0.12	0.25	0.34
	7	90.10	84.58	98.80	3.41			
	14	95.05	87.70	95.58	3.08			
Albumin (g L^−1^)	0	30.05	28.80	30.63	0.49	0.66	0.44	0.88
	7	30.68	29.90	32.75	0.61			
	14	29.05	29.93	29.45	0.65			
TP ^7^ (g L^−1^)	0	78.35	77.60	86.43	3.21	0.05	0.55	0.12
	7	76.78	77.48	88.90	3.23			
	14	81.40	80.90	84.40	1.96			
AST ^2^ (U L^−1^)	0	20.43	20.73	21.06	1.01	0.61	0.82	0.45
	7	19.86	22.41	19.67	0.77			
	14	20.46	20.46	18.91	0.94			
ALT ^3^ (U L^−1^)	0	49.32	50.77	53.57	2.82	0.44	0.41	0.55
	7	50.44	55.43	49.05	2.41			
	14	52.61	52.82	51.64	1.72			
GGT ^9^ (U L^−1^)	0	21.97	22.87	23.19	1.48	0.55	0.71	0.22
	7	21.87	23.74	20.02	1.33			
	14	20.87	22.61	23.48	1.01			
Lac ^11^ (mmol L^−1^)	0	0.40 ^a^	0.48 ^b^	0.54 ^b^	0.06	0.21	0.11	0.36
	7	0.50	0.40	0.55	0.04			
	14	0.50	0.60	0.48	0.03			
TAS ^10^ (mmol L^−1^)	0	1.09	1.08	1.11	0.02	0.52	0.23	0.15
	7	1.14	1.07	1.18	0.02			
	14	1.10	1.07	1.09	0.01			

^1^ Total cholesterol; ^2^ alanine aminotransferase; ^3^ aspartate aminotransferase; ^4^ high-density lipoprotein cholesterol; ^5^ low-density lipoprotein cholesterol; ^6^ triglycerides; ^7^ total protein; ^8^ non-esterified fatty acids; ^9^ gamma-glutamyl transferase; ^10^ total antioxidant status; ^11^ lactic acid; *p*–Value: TG for the effect of group, T for the effect of time, and TG × T for the interaction between group and time; ^a,b^—Means with different superscript letters differ significantly between groups (*p* < 0.05).

**Table 6:** Summary of alpha diversity metrics across experimental groups. Values are presented as mean ± SD for Observed ASVs, Chao1, Shannon, Simpson, and Inverse Simpson indices in the lime EO and (*R*)-(+)-limonene experiments across all groups and timepoints.

Experiment	Group	Observed ASVs (Mean ± SD)	Chao1 (Mean ± SD)	Shannon (Mean ± SD)	Simpson (Mean ± SD)	InvSimpson (Mean ± SD)
Lime EO	Lime EO T0	3812 ± 304	3814 ± 305	7.34 ± 0.06	0.998 ± 0.000	618.51 ± 90.91
	Lime EO T14	3932 ± 159	3935 ± 158	7.35 ± 0.08	0.998 ± 0.000	621.70 ± 80.29
	Ctrl Lime EO T0	3540 ± 152	3546 ± 150	7.28 ± 0.07	0.998 ± 0.000	613.43 ± 93.77
	Ctrl Lime EO T14	3658 ± 604	3664 ± 606	7.23 ± 0.24	0.998 ± 0.000	545.78 ± 154.20
(*R*)-(+)-Limonene	(*R*)-(+)-Limonene T0	2252 ± 386	2254 ± 386	6.89 ± 0.14	0.998 ± 0.000	456.90 ± 59.74
	(*R*)-(+)-Limonene T14	2487 ± 330	2494 ± 327	6.97 ± 0.20	0.998 ± 0.001	471.23 ± 145.07
	Ctrl (*R*)-(+)-Limonene T0	2354 ± 183	2358 ± 183	6.95 ± 0.10	0.998 ± 0.000	482.24 ± 75.91
	Ctrl (*R*)-(+)-Limonene T14	2638 ± 239	2642 ± 238	7.03 ± 0.12	0.998 ± 0.001	499.85 ± 103.12

## Data Availability

The raw data supporting the conclusions of this article will be made available by the authors on request.

## Supplementary Materials

The following supporting information can be downloaded at: https://www.mdpi.com/article/10.3390/molecules31142403/s1, Table S1. Significant differences in relative abundance of selected bacterial and archaeal taxa. Table S2. Detailed alpha diversity metrics. (**A**) Lime EO experiment; (**B**) (*R*)-(+)-Limonene experiment.
