# Supplementary material for: From Composition to Function: Lime Essential Oil (Citrus aurantifolia) and (R)-(+)-Limonene and Their Impact on Rumen Microbiota, Fermentation, Methane Emission and Blood Metabolic Parameters in Dairy Cows
Source: Molecules. 2026 Jul 8;31(14):2403. doi: 10.3390/molecules31142403 (PMC13413600; doi:10.3390/molecules31142403)
Supplement: Supplementary file 1 [file molecules-31-02403-s001.zip › molecules-4350758-supplementary.pdf]

Supplementary Table S1. Significant differences in relative abundance of selected bacterial and archaeal taxa. The table summarizes taxa showing statistically significant differences ($P < 0.05$) in relative abundance between experimental groups and/or timepoints in the Lime EO and (R)-(+)-Limonene experiments. Values are presented as mean ± SD.

Experiment	Family	Comparison	Relative abundance [%] (T0)	Relative abundance [%] (T3)	P-value (Kruskal-Wallis)
Lime EO	Halomonadaceae	Lime EO T0 vs. Lime EO T14	0.022 ± 0.014	0.005 ± 0.006	0,025
Lime EO	Bacteriovoracaceae	Ctrl Lime EO T14 vs. Lime EO T14	0.001 ± 0.001	0.006 ± 0.007	0,047
(R)-(+)-Limonene	Methanobacteriaceae	(R)-(+)-Limonene T0 vs. (R)-(+)-Limonene T14	0.455 ± 0.147	0.185 ± 0.052	0,021
(R)-(+)-Limonene	Gastranaerophilaceae	(R)-(+)-Limonene T0 vs. (R)-(+)-Limonene T14	0.287 ± 0.186	0.569 ± 0.134	0,021
(R)-(+)-Limonene	Elusimicrobiaceae	(R)-(+)-Limonene T0 vs. (R)-(+)-Limonene T14	0.000 ± 0.000	0.007 ± 0.002	0,022
(R)-(+)-Limonene	Paludibacteraceae	(R)-(+)-Limonene T0 vs. (R)-(+)-Limonene T14	0.025 ± 0.020	0.006 ± 0.005	0,047
(R)-(+)-Limonene	Comamonadaceae	Ctrl (R)-(+)-Limonene T0 vs. Ctrl (R)-(+)-Limonene T14	0.028 ± 0.022	0.162 ± 0.149	0,035
(R)-(+)-Limonene	Comamonadaceae	Ctrl (R)-(+)-Limonene T14 vs. (R)-(+)-Limonene T14	0.162 ± 0.149	0.017 ± 0.018	0,024

Supplementary Table S2. Detailed alpha diversity metrics. (A) Lime EO experiment; (B) (R)-(+)-Limonene experiment. Values represent per-sample alpha diversity indices including Observed ASVs, Chao1, Shannon, Simpson, and Inverse Simpson indices for all experimental groups and timepoints.

**A. Lime EO experiment**						
**Group**	**Sample**	**Observed ASVs**	**Chao1**	**Shannon**	**Simpson**	**InvSimpson**
**Lime EO T0**	LIM-7-1	3804	3805	7,29	0,998	597,78
	LIM-8-1	4058	4063	7,33	0,998	499,42
	LIM-11-1	4000	4002	7,44	0,999	702,61
	LIM-12-1	3386	3388	7,32	0,999	674,24
**Lime EO T14**	LIM-7-4	4022	4026	7,36	0,998	609,52
	LIM-8-4	4101	4103	7,44	0,999	711,87
	LIM-11-4	3752	3757	7,24	0,998	519,39
	LIM-12-4	3851	3852	7,35	0,998	646,02
**Ctrl Lime EO T0**	CTRL-1-1	3641	3645	7,34	0,998	660,76

	CTRL-6-1	3549	3557	7,24	0,998	588,16
	CTRL-9-1	3649	3651	7,19	0,998	494,56
	CTRL-10-1	3322	3331	7,33	0,999	710,22
**Ctrl Lime EO T14**	CTRL-1-4	3616	3624	7,17	0,998	494,48
	CTRL-6-4	4316	4325	7,52	0,999	764,24
	CTRL-9-4	2866	2869	6,94	0,998	403,02
	CTRL-10-4	3835	3837	7,28	0,998	521,40
**B. (R)-(+)-Limonene experiment**						
**Group**	**Sample**	**Observed ASVs**	**Chao1**	**Shannon**	**Simpson**	**InvSimpson**
**(R)-(+)-Limonene T0**	LR-2-1	2702	2704	7,05	0,998	533,32
	LR-3-1	1933	1936	6,78	0,998	450,97
	LR-4-1	2445	2447	6,95	0,998	455,90
	LR-5-1	1927	1927	6,77	0,997	387,40
**(R)-(+)-Limonene T14**	LR-2-4	2406	2414	7,04	0,998	560,79
	LR-3-4	2972	2974	7,21	0,998	627,65
	LR-4-4	2321	2325	6,83	0,997	362,34
	LR-5-4	2248	2261	6,79	0,997	334,14
**Ctrl (R)-(+)-Limonene T0**	S-1-1	2529	2532	7,01	0,998	519,96
	S-6-1	2355	2363	7,04	0,998	615,32
	LR-1-1	2380	2385	6,91	0,998	432,92
	LR-6-1	2482	2485	7,00	0,998	454,82
	K-3-1	2008	2010	6,76	0,998	403,05
	K-4-1	2369	2372	6,95	0,998	467,39
**Ctrl (R)-(+)-Limonene T14**	S-1-4	2403	2409	6,97	0,998	515,67
	S-6-4	3058	3059	7,18	0,998	602,53
	LR-1-4	2628	2631	7,10	0,998	513,16
	LR-6-4	2588	2597	6,99	0,998	501,22
	K-3-4	2725	2728	7,10	0,998	562,35
	K-4-4	2427	2430	6,85	0,997	304,17
